# Is autophagy associated with diabetes mellitus and its complications? A review

**DOI:** 10.17179/excli2018-1353

**Published:** 2018-07-24

**Authors:** Debalina Bhattacharya, Mainak Mukhopadhyay, Maitree Bhattacharyya, Parimal Karmakar

**Affiliations:** 1 Department of Biochemistry, University of Calcutta, Kolkata-700019; 2 Department of Life Science and Biotechnology, Jadavpur University, Kolkata-700032; 3 Department of Biotechnology, JIS University, Agarpara, Kolkata-700109

**Keywords:** type 2 diabetes mellitus, T2DM, autophagy, retinopathy, cardiomyopathy, nephropathy

## Abstract

Diabetes mellitus (DM) is an endocrine disorder. In coming decades it will be one of the leading causes of death globally. The key factors in the pathogenesis of diabetes are cellular injuries and disorders of energy metabolism leading to severe diabetic complications. Recent studies have confirmed that autophagy plays a pivotal role in diabetes and its complications. It has been observed that autophagy regulates the normal function of pancreatic β cells and insulin-target tissues, such as skeletal muscle, liver, and adipose tissue. This review will summarize the regulation of autophagy in diabetes and its complications, and explore how this process would emerge as a potential therapeutic target for diabetes treatment.

## Introduction of Autophagy

The term autophagy (means “self-eating”) was first depicted by Duve and Wattiaux in 1966[14]. This is referred to as a bulk degradation process which involved in the clearance of damaged proteins and organelles and is an evolutionarily conserved phenomenon from yeast to mammals. The autophagic pathway can be induced by stress, such as deprivation of nutrients or growth factors, hypoxia; as well as oxidative stress, pathogen infection and also by physical exercise. Autophagy plays an integral part in retaining cellular homeostasis by reutilizing intracellular energy assets in response to nutrient starvation (Huber et al., 2012[24]), and remove cytotoxic proteins and damaged organelles under adverse stress conditions (Kroemer et al., 2010[36]; Rabinowitz and White, 2010[55]). The process of cellular autophagy can be differentiated into three types: macroautophagy, microautophagy and chaperone-mediated autophagy (CMA). Macroautophagy (hereafter termed as autophagy) is the most pertinent pathway involving multistep processes with several vesicular fusion events (Barlow and Thomas, 2015[3]).

**Figure 1:**
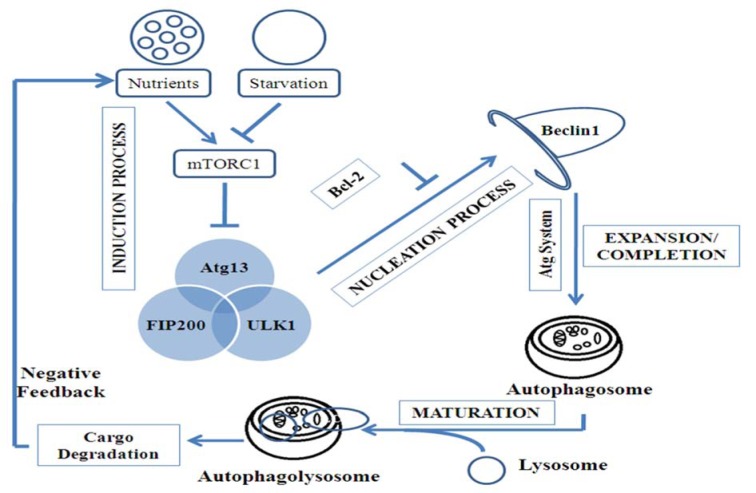
Step wise signaling of autophagy. There are four steps in autophagic process: induction, nucleation, expansion/completion and maturation

Autophagy commences by means of the formation of double-membrane vesicles, recognized as autophagosomes that swallow up cytoplasmic components. Autophagosomes combine with endosomes to form intermediate amphisomes. Amphisomes fuse with the lysosome to form autolysosomes. Autolysosomes are enriched with hydrolytic enzymes that degrade the autophagic cargo and release the degraded component into the cytoplasm of the cell for reuse (Figure 1(Fig. 1)). 

### The regulation of autophagy machinery

**Figure 2:**
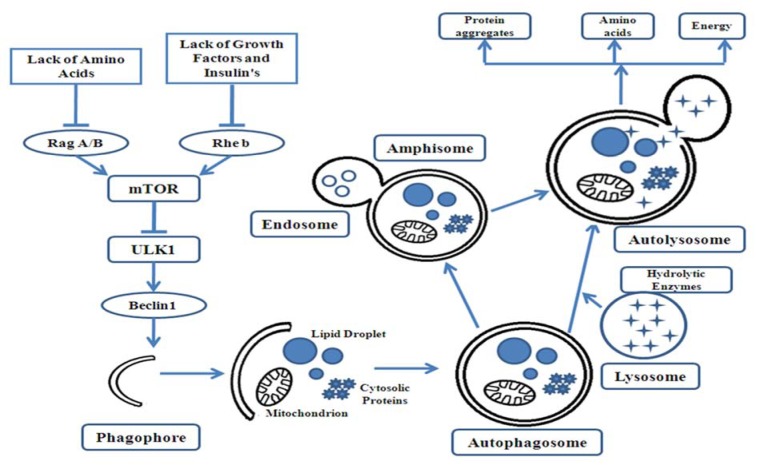
Autophagosome formation and degradation in type 2 diabetes

Autophagy could be regulated by several factors such as amino acids, insulin or protein sensors (Moruno et al., 2012[50]). The process of autophagy starts with the formation of an autophagosome, which can originate from the endoplasmic reticular (ER) membranes (Figure 2(Fig. 2)). This process has four key steps: initiation, nucleation, elongation, and closure (Liang et al., 1999[41]; Mizushima and Komatsu, 2011[47]). Each step of autophagosome formation is firmly synchronized by proteins encoded by autophagy-related genes (Atgs). Till date, more than 30 Atgs have been revealed in yeast and shown to have important roles in autophagy. Many of these genes have mammalian homologs. Autophagy is commenced with the activation of the unc-51-like kinase 1 (Ulk1) complex. Ulk1-mediated phosphorylations of Atg13 and FIP200 are necessary to set off autophagy. Phagophore nucleation is conditional on a Beclin1 (Atg6 in yeast), or class III phosphatidylinositol 3-kinase (PI3K) complex (Sin-Hye et al., 2017[64]; Zientara-Rytter and Subramani, 2016[82]). Autophagosome elongation and closure process involve two ubiquitin-like conjugation types of machineries: Atg12 and light chain 3 (LC3), the mammalian ortholog of yeast Atg8. The Atg12-Atg5 conjugate, which forms the Atg12-Atg5-Atg16 complex, comes up with the stimulation and localization of the LC3 conjugation. The cytosolic isoform of LC3 is LC3-I. It is conjugated to phosphatidylethanolamine through two successive ubiquitination-like reactions. This reaction is catalyzed by the E1-like enzyme Atg7 and the E2-like enzyme Atg3, forming membrane bound LC3-II. Therefore, LC3-II development is documented as a marker for the formation of autophagosomes in cell and animal systems (Kameyama et al., 2017[29]; Yao et al., 2017[76]). Next, the autophagosomes fuse with lysosomes to form autolysosomes so that the autophagosome containing macromolecules can be degraded by lysosomal enzymes. Protein p62, also known as sequestosome 1 (SQSTM1), confines to autophagosomes by interacting with LC3 and is time after time degraded by the autophagy-lysosome system (Fujimoto et al., 2017[18]). ULK1 is suppressed by mTORC1, a key negative regulator of autophagy, under nutrient-rich conditions (Pattingre et al., 2008[52]). mTORC1 is inhibited upon the initiation of autophagy, but in the intervening time, AMPK (AMP-activated protein kinase) can be activated. Moreover, dephosphorylation of ULK1 by mTORC1 inhibition or ULK1 phosphorylation by AMPK activates ULK1 (Jung et al., 2008[28]; Shang and Wang, 2011[61]). 

## The Regulation of Autophagy in Diabetes and its Complications

### Autophagy and pancreatic beta cells

The pancreatic beta cells are the major glucose regulatory cells in pancreatic islets which regulate the insulin secretions. The glucose level rises abruptly when these cells function abnormally either due to programmed cell death or by ER stress. Additionally, impairment of the autophagic pathway in pancreatic β cells also results in the development of type 2 diabetes. Recently it was revealed that “Constitutive” autophagy has a role in regulating the organization and function of pancreatic islet β-cells. For instance, β cell-specific deletion of Atg7 in type 2 diabetic mice leads to the malfunctioning basal autophagy, structural anomalies of islets and reduced insulin secretion. It has also been reported that the β cells derived from Atg7 knockout mice contains large inclusion bodies comprising ubiquitin and overexpressed p62, with reduced cell mass due to amplified apoptosis (Chen et al., 2011[10]). Recently, the research in diabetes depicts that a significant decline in pancreatic β cell populations is the major cause of the decrease in insulin production in T2D. In an earlier report, Butler et al. (2003[6]) have observed that β cell mass was significantly abated when pancreatic samples from autopsies of diabetic patients were compared with samples from non-diabetic subjects. Autophagy plays a decisive role in islet homeostasis and a compensatory boost of β cell mass in response to a fat-enriched diet (Ebato et al., 2008[15]; Jiang et al., 2014[27]; Marrif and Al-Sunousi, 2016[43]). Several studies have suggested that PINK1/PERKIN pathway plays a pivotal role in mitochondrial quality control in obese and T2DM. In skeletal muscle tissues of obese and T2D patients, transcription of PINK1 level is suppressed suggesting that mitochondrial quality control via PINK1/PERKIN pathways is reduced (Scheele et al., 2007[59]). Furthermore, impaired PARKIN function may damage pancreatic β cells and which in turn culminates in the insulin deficiency. At the onset of diabetes, the tumor suppressor protein p53 accumulates in the cytosol of mouse β-cells and inhibits mitophagy via the PERKIN pathway. Mice deficient in p53 have restored mitophagy and are resistant to β-cell reduction brought about by the streptozotocin (STZ) (Hoshino et al., 2014[22]).

Studies conducted at the cellular level in pancreatic β cells depicts that the autophagy performs a climactic role and for this, the pathway might comprise of the LC3, PKC (protein kinase C), JNK and others. It has been depicted that autophagy performs an imperative function in shielding the β cells against hIAPP oligomerization. Treatment of β cells with human islet amyloid polypeptide (hIAPP) amplifiers autophagosome development and inhibition of autophagy enhances the cytotoxic effects of hIAPP (Shigihara et al., 2014[63]). Furthermore, a combination of β-cell-specific autophagy deficit and hIAPP knock-in results in considerable reduction of glucose tolerance, while exclusively hIAPP knock-in has zero effects on β cell function (Costes et al., 2014[11]; Kim et al., 2014[33]; Shigihara et al., 2014[63]; Morita et al., 2011[49]). This diabetic phenotype can be partially reversed by the pharmacological advancement of autophagy and it curtails the accumulation of toxic hIAPP oligomers (Shigihara et al., 2014[63]; Kim et al., 2013[32]). Therefore, autophagy is found to be a vital process for the detoxification of toxic hIAPP oligomers. Recent studies envisaged that VAMP7 is a SNARE protein which mediates specific membrane fusions in intracellular trafficking and also modulates the development of autophagosome formation. However, its function in pancreatic β-cells is not yet explored (Aoyagi et al., 2016[1]). In a recent study, Aoyagi and colleagues (2016[1]) depicted the role of VAMP7 in regulating autophagy to uphold mitochondrial superiority and the release of insulin in response to the pathological stress in the β-cells.

### Autophagy and adipose tissue

Autophagy in adipose tissue (AT) has also been explored in connection to type 2 diabetes as an additional key target of insulin. The formation of mature white adipocytes with unique features needs drastic cytoplasmic reorganization. Nowadays the connection between autophagy and adipogenesis has been developed. Massive autophagy is activated during adipocytes differentiation of primary mouse fibroblast. Autophagy is obligatory for lipid storage, thereafter, Atg 5 and Atg 7 is essential for the segregation of white and brown adipocytes (Zhang et al., 2009[80]; Goldman et al., 2010[20]). Autophagy-deficient MEFs (atg7−/−, atg5−/−) exhibit noticeably reduced efficiency in adipogenesis (Baerga et al., 2009[2]). Research shows that if Atg7 was knocked out from the mice, it shows some unusual characteristics like the mice are lean, have improved metabolic rate, and are resistant to the development of obesity. These mice also established the conversion pathway of white AT into the brown AT, which leads to better glucose utilization, high rate of β-oxidation, and enhanced insulin sensitivity. Autophagy has been known to increase the expression of peroxisome proliferator-activated receptor (PPAR) γ, the key regulator of adipocyte differentiation and adipogenesis (Zhang et al., 2012[81], 2013[78]). In some cases, it was observed that animal with skeletal muscle-specific deletion of Atg7 results in impaired adipogenesis, suggesting the phenomenon of the crosstalk between autophagy and adipogenesis (Kim et al., 2013[32]; Stienstra et al., 2014[65]). The disordered physiological process of type 2 diabetes is directly coupled with increased autophagy in AT. Kosacka and colleagues examined the occurrence of autophagy in AT of type 2 diabetes (T2DM) patients in comparison to obese and lean individuals without diabetes (Kosacka et al., 2015[35]). It was observed that autophagy is up-regulated in type 2 diabetes. Additionally, autophagy makes AT inflammation and secretion of a pro-inflammatory adipokine. On the whole, functional autophagy supports adipocyte development and differentiation. Therefore, any impairment in the operation of autophagy may affect AT mass and homeostasis.

### Autophagy and diabetic cardiomyopathy

Diabetic cardiomyopathy (DCM) provides impetus to diabetes driven morbidity and mortality. Two-thirds of diabetic patients expire from heart disease or stroke globally. This has drawn attention to treating cardiovascular complications. Diabetic cardiomyopathy is one of the major complications of T2DM. It is characterized by reducing cardiomyocyte contractility, apoptosis, mitochondrial pathology, and dysfunction (Tate et al., 2017[67]) that develops in diabetic patients without coronary artery disease, hypertension or any other diseases. T2DM patients, with enhanced HbA1c (glycated hemoglobin) levels, have a 30 % higher chance of heart failure than nondiabetic individuals. Animal studies also demonstrated that diabetes can directly impair cardiac structure and function. Streptozotocin (STZ)-induced diabetic animals showed a reduced cardiac mass, myocardial hypertrophy, and cardiac fibrosis (Boudina and Abel, 2007[5]). Autophagy plays a vital role in the maintenance of normal heart structure and function by blocking the accumulation of dysfunctional organelles and cytotoxic protein aggregates. Studies concerning myocardial autophagy in T2D are very paltry and results are quite incongruent. Some of the studies reported that cardial autophagy can be unaltered (Lancel et al., 2012[39]), diminished (Sciarretta et al., 2012[60]; Xu et al., 2013[74]) or even enhanced (Mellor et al., 2011[44]) in T2DM. Several studies have shown that autophagosome accumulates in the hearts of diabetic mice (Ouyang et al., 2014[51]; Mellor et al., 2010[45]; Russo et al., 2012[58]) due to both increased formation and reduced lysosomal degradation (Mellor et al., 2010[45]; Wang et al., 2014[70]). Diabetic mouse models were prone to cardiac dysfunction by inhibiting autophagic flux with a lysosomal inhibitor (Mellor et al., 2010[45]). Various studies have associated mTORC1 activity to the development of diabetic cardiomyopathy. Volkers and colleagues (2014[69]) envisaged that PRAS40-mediated mTORC1 inhibition prevents the development of diabetic cardiomyopathy in the diabetic mice model (Volkers et al., 2014[69]). Rapamycin, a potent inhibitor of mTOR and inducer of autophagy, augments cardiac function in db/db type 2 diabetic mice (Das et al., 2014[13]). Conversely, enhancement of autophagic flux with resveratrol ameliorates diabetic cardiomyopathy (Wang et al., 2014[70]). Kubli and Gustafsson in 2014[37] reported that the risk of diabetic cardiomyopathy development is reduced by improving cardiac autophagy in type 2 diabetic patients. Pei and colleagues in 2015[53] have exhibited the ameliorative effects of desacyl ghrelin on cardiac dysfunction, cardiac fibrosis, and cellular autophagy in a T2DM mouse model. They demonstrated that desacyl ghrelin protects the heart against cardiac dysfunction by inducing cardiac autophagic pathway via the pro-survival cellular AMPK/ERK1/2 and inhibiting excessive collagen deposition. Zhang and colleagues in 2016 showed that early administration of trimetazidine could improve diabetic cardiomyopathy by inhibiting myocardial fibrosis, reducing cardiomyocyte apoptosis, and enhancing autophagy (Zhang et al., 2016[79]). In another study, Sun and colleagues (2016[66]) investigated the potential role and mechanism of Lin28a in myocardial dysfunction in diabetic mice. Lin28a protects against diabetic cardiomyopathy through PKA/ ROCK2 dependent pathway. Therefore, Lin28a can act as a potential antidiabetic drug in future.

### Autophagy and diabetic nephropathy

Diabetic nephropathy (DN) has surfaced as a prime cause of end-stage renal disease and is culminating into a grave global health hazard. DN is characterized by podocyte loss due to its detachment from the glomerular membrane (GBM). Earlier evidence has demonstrated that endoplasmic reticulum (ER) stress may contribute to the development of DN (Cunard and Sharma, 2011[12]; Cao et al., 2016[8]). Defects in autophagy accelerate the irreparable progression of DN in mice (Huber et al., 2012[24]). However, the role of ER stress and autophagy in DN has not been vividly depicted (Kume and Koya, 2015[38]). The functional roles of autophagy in kidney have been intensely investigated. For example, autophagy has been reported to play a pivotal role in renoprotection roles during both normal aging and after acute kidney injury in animal models (Huang et al., 2016[23]). It has been reported that podocytes and proximal tubular cells are the main cells affected in DN. In addition, several studies have accounted many renoprotective agents that induce autophagy for the treatment of DN in mice models. Cao and colleagues (2016[8]) investigated the role of ER stress inhibitors ursodeoxycholic acid (UDCA) and 4-phenylbutyrate (4-PBA) in the treatment of DN in db/db mice. They revealed that ER stress has an important role in the development of DN, and UDCA or 4-PBA treatment may emerge as a potential novel therapeutic approach for the treatment of DN.

Huang et al. (2016[23]) elucidated the role of KCa3.1 in human proximal tubular cells (*in vitro*) and in a mouse model of DN to study the underlying cellular mechanism of tubular autophagy. They envisaged that diabetes inhibited tubular autophagy was mediated by blocking of KCa3.1. This, in turn, was regulated by the PI3K/Akt/mTOR signaling pathway inactivation (Huang et al., 2016[23]). Ma et al. (2016[42]) investigated the mode of action of RSV-mediated protection against DN in diabetic rats. In addition, they focused on the role of NAD-dependent deacetylase sirtuin (Sirt1) in the regulation of autophagy. Xu and colleagues in 2016[75] reported that autophagy is suppressed under low insulin sensitivity of podocytes but rapamycin, a mTOR specific inhibitor, reverses the effect of podocytes by upregulating autophagy via attenuation of insulin resistance.

### Autophagy and diabetic retinopathy

The diabetic retinopathy (DR) is one of the most predominant complications associated with diabetes (Kaul et al., 2013[30]; Blake and Trounce, 2014[4]) and an overriding cause of blindness worldwide (Piano et al., 2016[54]). It occurs by the damage to the blood vessels of the light-sensitive tissue of the retina (Gaucher et al., 2007[19]; Piano et al., 2016[54]). Diabetes mellitus enhances inflammation, advanced glycation end products (AGEs) and oxidation of proteins including LDL. Fu and colleagues demonstrated that the oxidative stress and ER stress are induced by regulating the extravagated LDL, and are implicated in the pericyte loss involved in the case of DR (Fu et al., 2012[17]). In diabetic retinopathy (DR), stimulation of death receptors such as Fas and TNFα, mitochondrial damage caused by oxidative stress and endoplasmic reticulum (ER) stress are the major factors that set off apoptosis resulting in the cell damage.

Pericyte cells are indispensable for retinal capillary structure and function, and the loss is an early characteristic of DR. Autophagy aggravates viability of pericytes under mild stress, but results in their death under excessive stress. The proteins related to autophagy are constitutively expressed in the eye. Extensive studies have revealed that abnormal autophagy is a fundamentally important pathological feature of diabetic retinopathy. Additionally, the intricate networks between autophagy and apoptosis are instrumental in furnishing the degree of cellular apoptosis and the promotion of DR (Piano et al., 2016[54]; Rosa et al., 2016[57]). Fenofibrate is essential, a peroxisome proliferator-activated receptor (PPAR), an agonist currently used to bring down the levels of serum lipids. Miranda et al. (2012[46]) looked into the effect of fenofibric acid in human RPE cell line and they established that FA exhibits a bifold protective effect in RPE by down-regulation of stress-mediated signaling as well as by the induction of autophagy and survival pathways (Miranda et al., 2012[46]; Keech et al., 2007[31]). 

### Autophagy and diabetic neuropathy

**Figure 3:**
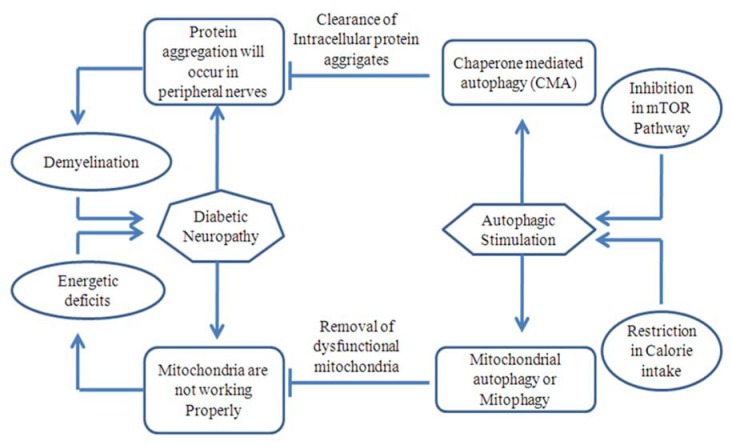
Hypothesis depecting autophagy activation in diabetic neuropathy

Diabetic neuropathy is the most common complication associated with diabetes mellitus. Hyperglycemia can injure nerve cells throughout the body. Incidences of diabetic neuropathy have very high prevalence (50-60 %) in patients associated with both types of diabetes. Diabetic neuropathy can be mediated through enhanced ROS generation, improper bioenergetics supply, apoptosis, hypercholestromia, dysfunction of mitochondria and endoplasmic reticulum, ischemia/hypoxia,nuclear factor kappa B (NF-κB)-directed neuro-inflammation, accumulation of damaged proteins and organelles in neurons and glial cells (Callaghan et al., 2012[7]; Cashman and Höke, 2015[9]; Feldman et al., 2017[16]; Gonçalves et al., 2017[21]). The progression and pathophysiology of neuropathy in impaired type 2 diabetes (T2DM) is poorly understood, especially in relation to autophagy (Figure 3(Fig. 3)). 

Autophagy can clear the protein aggregates and damaged organelles and helps to protect neurons from demyelination and bioenergetic crisis respectively (Gonçalves et al., 2017[21]; Renna et al., 2010[56]; Towns et al., 2005[68]). Recent studies have reported that macroautophagy is impaired in T2DM. Li et al. in 2017 observed that autophagy was reduced in the hippocampus of diabetic mice with hyperglycemia, whereas it was unchanged in mice without hyperglycemia (Li et al., 2017[40]). Autophagy is directly inhibited by high glucose in mouse primary hippocampal neurons. Moreover, autophagy has a protective role in high-glucose-induced neurotoxicity. Li et al. also reported that autophagic flux was suppressed by high glucose due to impaired autophagosome synthesis by GFP-LC3 analysis (Li et al., 2017[40]).The reason behind the reduction of autophagy was due to NO production under high glucose conditions. Finally, they conclude that autophagy impairment mediated by S-nitrosation of ATG4B leads to neurotoxicity in response to hyperglycemia.

In a very recent article, Moheseni et al. have investigated whether there is an association between the autophagic process and sural nerve fiber pathology (Mohseni et al., 2017[48]), so they have studied the sural nerve physiology and ultrastructure morphology at baseline and 11 years follow-up of normal glucose tolerance (NGT) and T2DM. Interestingly, they observed T2DM patients have lower nerve amplitude compared to NGT at baseline. Therefore autophagy occurs in sural nerves and can be affected by T2DM. As there is no FDA approved therapy available for DN, extensive research has developed for the synthesis of different therapeutic agents for DN. Quercetin is widely known as an antioxidant, anticancer, antidiabetic immunoregulatory agent. Recent studies have revealed that quercetin increased the proliferation of glial cells that were inhibited by high concentrations of glucose through autophagy induction (Kim et al., 2013[32]; Shi et al., 2013[62]; Wu et al., 2012[73]; Wang et al., 2011[71]). In a recent article, Inceoglu et al. in 2017[26] identified Epoxy Hydrolase, a non-channel, non-neurotransmitter therapeutic target for neuropathic pain of diabetic neuropathy. These sEH degrades the natural analgesic lipid mediators Epoxy fatty acids (EpFAs) through activation of endoplasmic reticulum (ER) stress. Neuropathic pain is blocked when EpFAs are stabilized by inhibiting sEH in diabetic animals, but the mechanism of diabetic neuropathy is poorly understood.

SirT1 (Sirtuin 1) is an important metabolic sensor. It responds to changes in the cellular NAD+/NADH ratio. It was proposed that SirT1-mediated FOXO3a signaling reverse diabetic memory-induced epigenetic changes (Ido, 2016[25]). Therefore SirT1 inactivation may be one of the reasons for the pathogenesis of insulin resistance and T2DM associated vascular complications. Recently Yerra et al. in 2017 have investigated the role of SirT1 activation by Isoliquiritigenin (ILQ) on AMP kinase (AMPK) and peroxisome proliferator-activated receptor gamma coactivator 1-alpha (PGC-1α) signaling in peripheral nerves of diabetic rats and in high glucose (30 mM)-exposed neuro2a (N2A) cells (Yerra et al., 2017[77]). ILQ, the known antioxidant, and the SirT1 activator are recently studied for its antidiabetic activity (Watanabe et al., 2016[72]). Diabetic rats and N2A cells, exposed to high glucose showed low expression of SIRT1 with a reduction in mitochondrial biogenesis and autophagy. Upon administration of ILQ in diabetic rats and as well as high glucose-exposed N2A cells showed significant SIRT1 activation with a concurrent increase in mitochondrial biogenesis and autophagy (Yerra et al., 2017[77]).

## Conclusions and Perspective

Autophagy is an important regulatory signaling pathway for type two diabetes mellitus. Autophagy induced by cellular stress could affect many tissues, glucose or lipid metabolism and secretion of insulin. Therefore, these conditions might lead to various chronic diseases such as diabetes complications (Figure 2(Fig. 2)). T2DM itself may not be such a serious problem. But the continuing tissue damage could lead to cellular injuries and homeostasis disruption. Autophagy induction can be determined by different protein signals, such as mTORC1, LKB1, ULK and LC3, AMPK, sirT1 etc. The crosstalk between autophagy and most important protein kinase AMPK might be a possible therapeutic target. In future research, blocking other regulatory pathways of autophagy-like mTORC1 might be significant, including AMPK upstream and downstream regulations. Nowadays, it has been observed that medicine, therapeutic approaches, and pathogenesis may tend to vary from person to person because of their genetic constituents. Therefore, autophagy can be used as a significant therapeutic approach by combining genetic and protein levels in AMPK for diabetes and its complications.

## Acknowledgement

We greatly acknowledge Dr. DS Kothari postdoctoral fellowship scheme of UGC, Government of India for funding this research.

## Conflict of interest

We have no conflict of interest.

## Ethical approval

This review article does not contain any studies with human participants or animals performed by any of the authors.
